# Reduction in self-reported influenza-like-illness in school children and household members following influenza vaccine administration – a cohort study, Israel, 2016–7

**DOI:** 10.1186/s13584-021-00478-6

**Published:** 2021-07-05

**Authors:** Noa Shviro Roseman, Natalya Bilenko, Rivka Sheffer, Zohar Mor

**Affiliations:** 1 Tel Aviv Department of Health, Ministry of Health, Tel Aviv, Israel; 2 School of Public Health, Ben Gurion University of the Negev, Beer Sheva, Israel; 3 School of Health Sciences, Ashkelon Academic College, Ashkelon, Israel

## Abstract

**Background:**

Second-grade pupils in Israel have been vaccinated against influenza since the winter of 2016–2017. This study aims to appraise the rate reduction of seasonal influenza vaccine among vaccinated children and their household members, and that of the vaccinated cohort and their household members.

**Methods:**

This retrospective cohort study was performed in winter 2016–2017 in Tel-Aviv District, Israel and compared second-grade pupils who were vaccinated at school, with third-grade pupils- who were not vaccinated at school. Parents in nine schools were asked to report prior vaccination against influenza and influenza-like illness (ILI) of their children and other household members. Rate reduction was defined as [(ILI among unvaccinated) – (ILI among vaccinated)] / (ILI among vaccinated) (%).

**Results:**

Of 527 participants, 359 (68.1%) were unvaccinated and 168 (31.9%) vaccinated. Unvaccinated children reported more ILI compared with vaccinated children (19.5% vs. 7.7%), yielding a rate reduction of 60.5%. Unvaccinated children also had a greater number of physicians’ visits and missed school days (35.7% vs. 14.9 and 42.9% vs. 25.6%, respectively). The rate of ILI among household members of unvaccinated children was 34.5%, compared with 25.0% among household members of vaccinated children.

The vaccinated cohort (defined as all children in second grade) reported less ILI compared with the unvaccinated cohort (defined as all children in third grade), with a rate reduction of 44.6%. Pupils of the unvaccinated cohort were more likely to miss school days (42.1% vs. 32.0%, respectively), and a higher rate of ILI was reported among household members of the unvaccinated cohort (35.4% vs. 27.3%, respectively).

**Conclusion:**

Influenza vaccine administered in school setting reduced ILI among the vaccinated cohort and their household members by 60.5 and 27.5%, respectively, compared with the unvaccinated cohort. Expansion of the vaccination program in a school setting increased the public health benefit of influenza vaccines among both school children and their household members.

## Introduction

Influenza virus belongs to the Orthmyxoviridae family and consists of three antigenic types: A, B, and C. Disease in humans is caused mainly by types A (dominated by subtypes H1N1 and H3N2) or B - lineages Victoria and Yamagata [1]. The virus is transmitted by droplets through coughing or sneezing, or by direct contact with a contaminated surface. The disease usually manifests with fever, chills, headache, malaise, widespread myalgia, dry cough, sore throat, nasal congestion, runny nose, vomiting and loss of appetite [1, 2]. The highest infection rate has been reported among school children, and secondary distribution may also occur in household members of the infected children [1]. Other age groups who are at risk for severe morbidity include people older than 65, individuals with immune suppression or chronic diseases such as diabetes, as well as pregnant women [3]. The burden of disease morbidity can be estimated by measuring influenza-like illness (ILI), which is defined as fever and cough. Both are the major symptoms of influenza. This symptomatic surveillance is used as a proxy to monitor the incidence of influenza without laboratory confirmation [4].

The influenza virus is more prevalent during autumn and winter in all strata of the population, infants to elderly. The average attack rate seasonal (non-pandemic) influenza is estimated at 20–30% in children and 5–10% in adults [5]. Severity of the disease varies from asymptomatic infections, to morbidity requiring hospitalization, to death [6]. Severity is usually greatest among the elderly and very young children. The hospitalization rate among children younger than 2 years of age ranges between 190 and 480 cases per 100,000 children [1].

For 2016–2017, the Advisory Committee on Immunization Practices of the US Centers for Disease Control and Prevention (CDC), and later the Israeli Ministry of Health (MoH) recommended influenza vaccination for the entire population over the age of 6 months who had no contraindications [5, 7]. In recent years, two inactivated vaccines were used in Israel. The first was a trivalent seasonal influenza vaccine, which included two subtypes of A and one B-type (either the Victoria or the Yamagata lineage). The second, a quadrivalent vaccine, included two subtypes of type A and two B-types (both Victoria and Yamagata lineage). In one meta-analysis study, the effectiveness of the trivalent inactivated vaccine among healthy children younger than 18 years of age was estimated to be 32.5–42.6% [8].

School vaccination has been shown to be effective in reducing morbidity among children and their household members [9]. In various schools over the United States, where seasonal influenza vaccine was offered for free, ILI or fever of the entire vaccinated cohort was reported to be lower by 23.1% compared with schools where seasonal influenza vaccines were not administered, ILI and fever among the parents was reduced by 27.3%, and fewer physician’s visits, limited use of medication, and less absenteeism days from school were reported [9].

The Israeli MoH changed its influenza immunization program in the winter of 2016–2017 and offered inactivated influenza vaccine in school to pupils in the second grade, in addition to retaining the recommendation of influenza vaccine for all individuals at the age of 6 months and above. Following this policy change, the aim of this study was to compare the rates of ILI between vaccinated pupils and their household members with that of non-vaccinated pupils. In addition, the study compared the rates of ILI between the entire vaccinated second-grade cohort and their household members with that of the third-grade cohort who were not vaccinated at schools, but could be immunized in the community, at their parent discretion. The results of this study were used by public health officials to appraise the rate reduction of ILI and to evaluate the effectiveness of influenza vaccines in primary school setting.

## Methods

This retrospective cohort study was conducted between December 2016 and April 2017, and included nine elementary schools in the Tel Aviv District in Israel. Study questionnaires were distributed among the parents of approximately 1500 children in second and third grades in those schools. All second grade pupils were offered influenza vaccination at school, while third grade pupils could be vaccinated at their parents’ discretion in the community. The parents were asked to respond anonymously to 14 questions regarding pervious influenza vaccine uptake, ILI symptoms among household members, absenteeism from school and work, and hospitalizations due to respiratory illnesses. ILI was defined in this study as fever > 37.5 °C and cough, which started between 14 days and up to 12 weeks following the day of vaccination at the school. Reports of ILI during the first fourteen days following the influenza vaccination were not included in this study, to avoid potential bias of adverse reactions due to the vaccine, which may be erroneously reported as ILI. An immunized individual in this study was defined as a pupil who received at least one dose of influenza vaccine during the 2016–2017 flu season. Household members were defined as family members who shared the same household with the pupil. Schools were included in this study if influenza immunization rates exceeded 50%.

Dependent variables in the study included the rate of ILI among second and third grade children and household members, and rate reduction, which was defined as [(ILI among unvaccinated) – (ILI among vaccinated)] / (ILI among vaccinated) (%).

ILI rates were compared between influenza-vaccinated children and non-vaccinated children, as well as between the vaccinated cohort with the non-vaccinated cohort. Sequential variables were compared by the Student’s *t*-test in cases where the distribution demonstrated a normal pattern and the Mann Whitney test otherwise. Categorical variables were compared by the Chi-square test. *P* values < 0.05 were considered statistically significant.

The influenza vaccine administered in 2016–2017 season in the school setting in Israel was Vaxigrip, an inactivated, split virion, vaccine by Sanofi Pasteur. The study was approved by the Institutional Review Board of the Israeli Ministry of Health (MOH 171–2016).

## Results

Of approximately 1500 questionnaires which were distributed to parents of second and third grade pupils in Tel Aviv, 543 (36.2%) were completed, of which 536 (98.7%) were available for statistical analysis. Of the entire sample, 133 (52.8%) of second grade pupils and 35 (12.7%) of third grade pupils were vaccinated for seasonal influenza during 2016–2017 winter season.

Unvaccinated children and their household members were less likely to be previously vaccinated against seasonal influenza during former winter seasons than vaccinated children and their household members (Table 1). A higher proportion of unvaccinated children reported ILI compared with vaccinated children, with rate reduction of 60.5%. The unvaccinated children also had a greater number of physicians’ visits and a higher number of missed school days. The household members of unvaccinated children also reported more ILI symptoms compared with household members of vaccinated children, with rate reduction of 27.5% (*p* = 0.03).

**Table 1:** Demographic and clinical characteristics of vaccinated pupils vs. unvaccinated pupils

Characteristic		Vaccinated (*N* = 168) N (%)	Unvaccinated (*N* = 359) N (%)	*P*
Grade (*N* = 527)	second	133/168 (79.2%)	119/359 (33.1%)	< 0.001
Gender (*N* = 524)	Male	86/166 (51.8%)	171/358 (47.8%)	0.4
Prior influenza vaccination (*N* = 523)		97/168 (57.7%)	122/355 (34.4%)	< 0.001
Vaccination of household members (*N* = 526)		94/167 (56.3%)	74/359 (20.6%)	< 0.001
Clinical manifestations during winter season	ILI	13/168 (7.7%)	70/359 (19.5%)	< 0.001
	Fever	32/168 (19.0%)	110/359 (30.6%)	0.006
	Cough	27/168 (16.1%)	126/359 (35.1%)	< 0.001
	Headache	40/168 (23.8%)	110/359 (30.6%)	0.1
	Malaise	34/168 (20.2%)	114/359 (31.8%)	0.007
Number of physician visits (*N* = 527)		25/168 (14.9%)	128/359 (35.7%)	< 0.001
School days lost (*N* = 527)		43/168 (25.6%)	154/359 (42.9%)	< 0.001
Emergency room or urgent medical care visit (*N* = 527)		6 (3.6%)	19 (5.3%)	0.5
Hospitalization (*N* = 527)		0 (0.0%)	2 (0.6%)	1.0
ILI symptoms among household members (*N* = 527)		42 (25.0%)	124 (34.5%)	0.03

ILI = Influenza Like Illness

Table 2 compares the entire group of second grade pupils with the entire group of third grade pupils. The third grade pupils (unvaccinated cohort) were more likely to report ILI compared with pupils of the second grade pupils (vaccinated cohort), with a rate reduction of 44.6%. In addition, pupils in the third grade were more likely to miss school days, and their household members reported more ILI compared with household members of the second grade pupils, with rate reduction of 22.9% (*p* = 0.05).

**Table 2:** Demographic and clinical characteristics of 2nd grade pupils (vaccinated cohort) vs. 3rd grade pupils (non-vaccinated cohort)

Character		Second grade (*N* = 256) N (%)	Third grade (*N* = 280) N (%)	*P*
Gender (*N* = 527)	male	135/250 (54.0%)	123/277 (44.4%)	0.3
Previous vaccination (*N* = 526)		116/252 (46.0%)	104/274 (38.0%)	0.06
Vaccination of household members (*N* = 528)		90/252 (35.7%)	78/276 (28.3%)	0.08
Clinical manifestations (*N* = 536)	ILI	29/256 (11.3%)	57/280 (20.4%)	0.005
	fever	61/256 (23.8%)	84/280 (30.0%)	0.1
	cough	63/256 (24.6%)	93/280 (33.2%)	0.03
	headache	75/256 (29.3%)	78/280 (27.9%)	0.8
	malaise	68/256 (26.6%)	82/280 (29.3%)	0.5
Physician visit (*N* = 536)		66/256 (25.8%)	90/280 (32.1%)	0.1
Number of children who missed school (*N* = 536)		82/256 (32.0%)	118/280 (42.1%)	0.02
Emergency room or urgent medical care visit (*N* = 536)		14/256 (5.5%)	11/280 (3.9%)	0.4
Hospitalizations (*N* = 536)		1/256 (0.4%)	1/280 (0.4%)	1.0
ILI symptoms among household members (*N* = 536)		70/256 (27.3%)	99/280 (35.4%)	0.05
Total vaccinated (*N* = 527)		133/252 (52.8%)	35/276 (12.7%)	< 0.001

## Discussion

The results found in this study are compatible with the upper range of vaccine effectiveness (VE) of 42–65.0%, which was demonstrated in Hong Kong and Sao Paulo, respectively [10, 11]. In the CDC report for seasonal influenza vaccine effectiveness in 2016–2017, adjusted vaccine effectiveness for all ages was 40%, and for age group 9–17 years vaccine effectiveness was 36% [12].

As many as 55% of second grade pupils in Israel were immunized against influenza in 2016–2017, higher than 21 and 24% coverage between 2015 and 2017 in the total population and among infants and children aged 6–59 months, respectively [13, 14]. This is consistent with the demonstration in Monroe County, New York that school-based vaccination may facilitate administration of vaccines and increase immunization coverage [15]. Reasons for higher immunization rates for school-based influenza vaccination may also include convenience for the parents, and perception of the vaccine as important since it is administered as part of universal mass vaccination program. Nevertheless, school-based immunization rate could have been even higher if more parents gave their consent to vaccinate their children.

Our finding that school-based influenza vaccination was associated with a rate reduction in ILI among household members is compatible with results of seasonal influenza vaccine effectiveness from other countries. A study from Italy demonstrated school based seasonal influenza vaccine effectiveness of 30% in prevention of respiratory tract infections for the household members [16]. Other examples of herd protection for older population and households following mass vaccination programs for children, include the success in pneumoccocal conjugate vaccine and hepatitis A vaccine [17, 18]. Vaccine effectiveness and herd protection could be cost saving both for the vaccinated child and the entire family, and the healthcare system. Cost savings may result from lower parents’ absenteeism from work due to lower morbidity among the parents and fewer school days lost in vaccinated children [19].

In our study, 56.3% of the household members of the vaccinated pupils reported they were vaccinated for seasonal influenza, compared with 20.6% of the household members of the unvaccinated pupils. A higher rate of vaccination of household members may reflect general willingness of a family to be immunized, and it is possible that school-based vaccination of children in the second grade encouraged their household members to get vaccinated themselves. It may also be that parents who already perceive the vaccine as important and approved their second-grade children to be vaccinated in school would be more supportive to immunize themselves.

The trivalent inactivated influenza vaccines which was used in schools in Israel consisted of two types A influenza and one type B. Among all influenza virus isolates in Israel in 2016–2017, type A specimens consisted of 97.9% of the total, while type B influenza consisted of only 2.1% of the total. Of the type A isolates, 99.8% were A(H3N2) serotype, which was included in the vaccine administered to schoolchildren in Israel [13]. In our study, 19.5% of the unvaccinated children reported ILI symptoms, compared with only 7.7% of the vaccinated children. As influenza vaccines are most effective when the antigens in the vaccine match those of circulating strains [20], the vaccine effectiveness reported in our study undoubtedly was influenced by the matching of the predominant circulating influenza virus strain A(H3N2) in Israel in winter 2016–2017 and the influenza virus types included in the vaccine that year.

Our study is subject to several limitations. First, a selection bias in possible, as the study was conducted in the Tel Aviv district in schools where vaccination rates were higher than 50%. In schools where the vaccination rate is lower, a lower rate reduction could be expected. In addition, only ~ 30% of the parents completed the questionnaires, which could limit the generalizability of the study. Second, although the questionnaires were completed anonymously, a reporting bias by the parents is possible. Third, the Israeli Ministry of Health recommended that children younger than nine years who were not previously vaccinated with seasonal influenza vaccine receive two doses of vaccine. Since the school vaccination program in Israel included just a single dose of seasonal influenza vaccine for the second grade, and our study questionnaire did not include any data regarding a second dose of the influenza vaccine, it is likely that some pupils did not receive the additional dose as recommended. That could have lowered the observed rate reduction in this study. Fourth, ILI in this study was recorded from parents’ self-reports rather than laboratory confirmation by polymerase chain reaction. Lastly, as the accurate timeframe between the vaccination and the onset of ILI was not available, vaccine effectiveness was not calculated and the results were presented by “ILI rate reduction”.

## Policy implications and conclusion

This study demonstrated that seasonal influenza vaccine administered in school setting in Israel reduced self-reported ILI among the vaccinated cohort and their household members. In 2017–2018 and 2018–2019, following the recommendation of Israel’s National Immunization Technical Advisory Group, the influenza vaccination program in the school setting in Israel has been further expanded to third and fourth grade children. It is expected that ILI rates will be reduced further, in parallel with the greater number of children who are vaccinated in schools.

## Data Availability

The datasets generated during and/or analysed during the current study available from the corresponding author on reasonable request.
